# The Influence of Co-Surfactants on Lamellar Liquid Crystal Structures Formed in Creams

**DOI:** 10.3390/pharmaceutics12090864

**Published:** 2020-09-11

**Authors:** Delaram Ahmadi, Najet Mahmoudi, Richard K. Heenan, David J. Barlow, M. Jayne Lawrence

**Affiliations:** 1 Institute of Pharmaceutical Science, King’s College London, Franklin Wilkins Building, 150 Stamford Street, London SE1 9NH, UK; delaram.ahmadi@kcl.ac.uk; 2 STFC ISIS Facility, Rutherford Appleton Laboratory, Chilton, Didcot OX11 0QX, UK; najet.mahmoudi@stfc.ac.uk (N.M.); richard.heenan@stfc.ac.uk (R.K.H.); 3 Division of Pharmacy & Optometry, School of Health Sciences, Stopford Building, University of Manchester, Oxford Road, Manchester M13 9PG, UK

**Keywords:** small-angle neutron scattering, creams, soft matter, colloidal systems, liquid crystalline structures

## Abstract

It is well-established that oil-in-water creams can be stabilised through the formation of lamellar liquid crystal structures in the continuous phase, achieved by adding (emulsifier) mixtures comprising surfactant(s) combined (of necessity) with one or more co-surfactants. There is little molecular-level understanding, however, of how the microstructure of a cream is modulated by changes in co-surfactant and of the ramifications of such changes on cream properties. We investigate here the molecular architectures of oil-free, ternary formulations of water and emulsifiers comprising sodium dodecyl sulfate and one or both of the co-surfactants hexadecanol and octadecanol, using microscopy, small-angle and wide-angle X-ray scattering and small-angle neutron scattering. We then deploy these techniques to determine how the structures of the systems change when liquid paraffin oil is added to convert them to creams, and establish how the structure, rheology, and stability of the creams is modified by changing the co-surfactant. The ternary systems and their corresponding creams are shown to contain co-surfactant lamellae that are subtly different and exhibit different thermotropic behaviours. The lamellae within the creams and the layers surrounding their oil droplets are shown to vary with co-surfactant chain length. Those containing a single fatty alcohol co-surfactant are found to contain crystallites, and by comparison with the cream containing both alcohols suffer adverse changes in their rheology and stability.

## 1. Introduction

Lamellar lyotropic liquid crystals have gathered much interest over the years as they form the highly structured continuous phases of semi-solid oil-in-water (o/w) emulsions that are present in pharmaceutical and cosmetic skincare formulations and also found across a range of processed foodstuffs and in veterinary preparations [1,2]. The emulsions formed from immiscible oil and water phases are thermodynamically unstable and, as a means to improve their stability, so-called emulsifiers are added to reduce the interfacial tension. The emulsifiers added are generally mixtures of amphiphilic molecules, typically an ionic or non-ionic surfactant mixed with one or more co-surfactant(s) such as fatty alcohols, fatty acids or monoglycerides [3,4,5]. It is the combination of surfactant(s) with co-surfactant(s) in the emulsifiers that is essential for the formation of a cream; the use of surfactant(s) alone is insufficient.

During the manufacture of creams, at high temperatures and at a high emulsifier concentration—beyond that needed to form an interfacial film at the oil droplet/water interface—the amphiphilic emulsifier molecules interact with the continuous phase and self-assemble as bilayers separated by regions of interlamellar water, thereby forming a lamellar liquid crystal phase [4,6]. When the system is then cooled to a temperature below the phase transition temperatures of the component amphiphiles, these liquid crystals swell to form an α-crystalline gel phase, where their hydrocarbon chains are hexagonally packed with the freedom for rotation about their long axes [4].

Friberg and co-workers [6,7,8] were among the first to propose that the presence of a lamellar liquid crystal phase in a water-oil-emulsifier system led to an increase in emulsion stability by forming a multilayer arrangement around the emulsion droplets, modifying the van der Waals interactions between the droplets, increasing their repulsion, and enhancing the viscosity of the formulation [2,9]. Friberg et al. [6,10] subsequently established that the lamellar liquid crystal structures existed independent of the emulsion by the successful separation of the lamellar “phase” from the emulsion through centrifugation. The structures of water-surfactant-co-surfactant systems have thus been widely studied as structural models for the continuous phases of creams.

When Eccleston subsequently expounded the gel-network theory of cream structure [4], her ideas were founded upon her findings obtained in small-angle X-ray diffraction experiments wherein she observed how the addition of a small amount of cetrimide surfactant led to the phenomenal swelling of a system comprising water and the commercially available mixture of hexadecanol and octadecanol [11]. Eccleston also examined the stability of this ternary system and its corresponding oil-containing cream when the hexadecanol/octadecanol mixture in the formulation was substituted by hexadecanol or octadecanol alone. Using rheology and differential scanning calorimetry (DSC), Eccleston found that the stability of these formulations was significantly compromised by comparison with the formulation containing equal amounts of hexadecanol and octadecanol, with the single alcohol systems eventually breaking down to form liquids. In the case of the hexadecanol formulation, the viscosity reduced progressively over time and the preparation broke down totally after a period of 2 weeks; with the octadecanol formulation, this breakdown occurred just 7 days post-preparation [11].

In our previous studies on creams based around Aqueous Cream B.P. [3,12] we successfully revealed the microstructure of a system comprising water, liquid paraffin oil, sodium dodecyl sulfate (SDS) surfactant and an equimolar mixture of hexadecanol and octadecanol as co-surfactants. In both the aqueous creams and their corresponding oil-free ternary systems, we found that an excess of emulsifier (10% w/w) gave rise to a lamellar gel network phase. The bilayers in the gel network were found to be composed primarily of the long-chain alcohol co-surfactants and with little, if any, SDS [13,14,15]. In the case of the creams, the bilayers were also found to contain small amounts of oil. In addition, we discovered structures present in the creams that had not been reported previously, with the SDS surfactant found to mix with one or both of the co-surfactants forming oblate ellipsoidal bicelles dispersed within the continuous aqueous phase. When the nature of the surfactant was changed as dodecyltrimethylammonum bromide (DTAB) or distearoylphosphatidylcholine (DSPC), we were surprised by the absence of DTAB/co-surfactant bicelles in the former system, and found that the latter contained unilamellar DSPC vesicles [12].

In the studies reported here, we extend our investigations on model creams [3,12], investigating formulations based around the UK licensed, British Pharmacopoeial product, Aqueous Cream B.P. In keeping with Eccleston’s studies [11,16], we explore the consequences of changing the added co-surfactant on the molecular structure, rheology and stability of the creams and their corresponding ternary systems and elucidate the role of the co-surfactant alcohols in the gel network formation. The formulations were varied to include a constant 1:5 mole ratio of surfactant to co-surfactant but with three variations in co-surfactant explored, with the co-surfactant taken either as an equimolar (1:1) mixture of hexadecanol and octadecanol, or hexadecanol (1:0) or octadecanol (0:1) alone. As in our previous studies, we also varied the emulsifier concentration of these systems so that they contained either 4% *w/w* or 10% *w/w*. The macroscopic properties of the creams were determined using bright-field and polarised light microscopy together with rheology measurements, and these were then related to the structures of the creams and their corresponding ternary systems which were determined at the nanoscale using small-angle and wide-angle X-ray scattering (SAXS and WAXS, respectively) and small-angle neutron scattering (SANS).

## 2. Materials

1-hexadecanol (cetyl alcohol; ReagentPlus^®^, 99% purity), 1-octadecanol (stearyl alcohol; ReagentPlus^®^, 99% purity), and sodium dodecyl sulphate (SDS) (Gas chromatography ≥98.0% purity) were purchased from Sigma-Aldrich (Gillingham, UK). Liquid paraffin was purchased from Merck and Co., Inc. (Darmstadt, Germany) All formulations were prepared using either ultrapure water (18.2 MΩ.cm obtained from Elga LabWater; High Wycombe, UK) or—for the purposes of the neutron scattering experiments—using an equivalent volume of D_2_O (99.9 atom % D), purchased from Sigma-Aldrich (Gillingham, UK).

## 3. Methods

Aqueous creams were prepared with water, liquid paraffin oil and either a 4% *w/w* or 10% *w/w* total concentration of emulsifier—a mixture comprising the surfactant, SDS and the co-surfactants, hexadecanol and octadecanol (compositions as detailed in Table 1). Whilst the mole ratio of surfactant:co-surfactant(s) was kept constant at 1:5, the added co-surfactants were varied in both the 4% *w/w* and 10% *w/w* emulsifier creams to include either a 1:1, 1:0 or 0:1 mixture of hexadecanol:octadecanol. The corresponding oil-free (ternary) versions of these creams were also prepared, adjusting the level of water in the formulation to account for the absence of oil in the preparations (see Table 1). Creams and ternary systems containing 4% and 10% *w/w* emulsifiers are henceforth referred to as 4% or 10% creams or ternary systems.

### 3.1. Method of Sample Preparation

Oily and aqueous phases were heated separately to around 353 K. Once at temperature, the surfactant was dispersed in the aqueous phase using a DrM Fundamix^®^ vibromixer and mixed at a moderate speed (setting 4; ~2400 revolutions per minute (rpm)) for three to four minutes. The oily phase was then transferred to the aqueous phase at around the same temperature and the sample was mixed at high speed (setting 6; ~3600 rpm) for a further three minutes. The mixture was then allowed to cool to room temperature, with continuous mixing, increasing the speed as the formulation became visibly more homogeneous.

### 3.2. Microscopy

Microscope slides were prepared by smearing a pin-tip amount of the one-day-old cream samples on the slide and making this as thin as possible by gently pressing down with a coverslip. The slides were then viewed under a Leitz Dialux 22 EB microscope fitted with a digital Zeiss AxioCam HRc camera (Oberkochen, Germany). Samples were analysed using a 40× magnification lens under both bright-field and polarised light. Bright-field images allowed for the oil droplet sizes to be analysed while images obtained under polarised light allowed for the detection of birefringence. Images of samples were recorded one-day post preparation at ambient temperature.

ImageJ [17] was used to analyse oil droplet sizes in the bright-field photomicrographs of the cream preparations. Images were first calibrated to have an appropriate scale (using an appropriate stage micrometre), then the droplets were selected according to their shape, and the areas of these shapes were measured, recorded and tabulated using the software’s built-in ruler function and measurement utilities. Each image was divided into an even number of grid squares and the grid squares then selected at random to provide sampling of ca. 400 droplets per image per sample. For each sample, at least four images were taken to account for each quadrant of the area occupied by the sample on the microscope slide.

Droplet areas (A) calculated using ImageJ were converted as droplet diameters (2√(A/π)) and the number frequency of the collated diameters fitted to a log-normal distribution using Origin© [18]. Cream samples were thus characterized by means of their mean droplet diameter and the log standard deviation on this diameter. Given the uncertainties on the sizes of oil droplets whose computed sizes fell below the resolution limit for visible light (200 nm), the number frequencies for these droplets were regarded with due caution.

### 3.3. Rheology

Measurements of viscosity against shear rate were made on the one-day-old cream samples (C4, C5 and C6, Table 1) using an ARES rheometer (TA instruments) with a parallel plate geometry (diameter 25 mm, gap 1 mm) at 298 K. The measurements involved transferring a spatula full of the cream onto the centre of the rheometer plate, lowering the plate until it touched the sample, removing any excess cream, and then allowing the sample to rest for three minutes. Viscosity measurements on the rested samples were then made in logarithmic mode, recorded over shear rates in the range 0.01–100 (s^−1^). All measurements were made in triplicate on one-day-old samples, and samples were stored at a constant temperature for up to one month. Further viscosity measurements were made at weekly intervals for six months or until the samples became too fluid for measurements to be performed.

A power-law model was applied to the linear portions the log-log viscosity vs. shear rate flow curves using the equation 𝜂 = K 𝛾^n−1^ where 𝜂 is the cream viscosity (Pa.s), 𝛾 is the shear rate (s^−1^), K is the consistency index (Pa.s^n^) and 𝑛 is the flow behaviour index [19]. For each formulation analysed, the slope of the log-log plot afforded the flow behaviour index, and the antilogarithm of the intercept on the ordinate in the log-log plot (where log(𝛾) = 0) yielded the viscosity at a shear rate 𝛾 of 1 s^−1^. The statistical significance of the differences observed in the viscosities of the cream formulations was assessed by means of a student’s *t*-test using Microscoft Excel^®^ (version 2013), accepting a significance level of *p* ≤ 0.05.

### 3.4. Small and Wide-Angle X-ray Scattering (SAXS and WAXS)

SAXS and WAXS measurements on the ternary systems and creams were made at room temperature on a Nano-inXider instrument (Xenocs, Sassenage, France) using a micro-focus sealed-tube Cu 30W/30 µm X-ray source (Cu K-α, λ = 1.54 Å) with samples loaded in a gel capsule holder. The SAXS and WAXS diffraction patterns (covering the respective *Q* ranges of 0.0045 Å^−1^ to 0.37 Å^−1^ and 0.3 Å^−1^ to 4.1 Å^−1^) were detected simultaneously using two Dectris Pilatus 3 hybrid pixel detectors. The periodicity of the lamellar phases seen in the SAXS profiles for the systems that gave rise to well-defined Bragg peaks and a baseline Porod scattering were determined by applying a simple Power law model to fit the underlying Q^−4^-dependent (Porod), and this was subsequently subtracted from the SAXS profiles to reveal the positions of the sharpened Bragg peaks more clearly.

The WAXS profiles were de-convoluted using LAMP [20] and the positions of the component peaks obtained were used to calculate their corresponding *d*-spacings as d = 2π/Q, where d is the in-plane lateral separation and Q, the scattering vector [9].

### 3.5. Small-Angle Neutron Scattering (SANS)

SANS measurements were made on one-day-old creams and their corresponding ternary systems as presented in Table 1, with all samples prepared with all components protiated in D_2_O. Samples were loaded in purpose-built cells as reported previously [3] and measurements were made on the LoQ instrument at the ISIS pulsed neutron source (STFC Rutherford-Appleton Laboratory, Harwell Oxford, United Kingdom). For each sample, the SANS measurements were recorded at 298, 305, 310 and 318 K (± 0.1 K) with the sample environment temperature controlled using a Julabo water bath [21]. LoQ uses neutrons of wavelength (λ) 2.2–10 Å, which are recorded at a 64 cm^2^ two-dimensional detector at a fixed distance of 4.1 m from the sample. The resulting SANS measurements provide for a momentum transfer Q (Q = 4πsin θ/λ, where θ is half the scattering angle) in the range 0.008 ≤ Q ≤ 0.22 Å^−1^.

Measured SANS data were processed using wavelength-dependent corrections to allow for the incident spectrum, detector efficiencies, and measured sample transmissions (as described in detail in Heenan et al. [22]. The SANS data were put on an absolute scale to give a profile of scattering intensity I(Q) as a function of Q, using the scattering from a standard sample (comprising a solid blend of protiated and perdeuterated polystyrene) in accordance with established procedures [23]. All scattering profiles were checked for multiple scattering by ensuring that the scattering profiles obtained with different linear ranges of wavelengths overlapped.

The SANS profiles for the 4% and 10% ternary systems were model-fitted using a para-crystalline lamellar stack model coded by Heenan (Model A1; personal communication) for use with the SasView package [24]. This model accounts for scattering from a para-crystalline lamellar stack taking into account 13 parameters, namely the sample background, the d-spacing of the bilayers within the lamellar stacks, the polydispersity on this d-spacing, the thickness of the bilayers, the polydispersity on the bilayer thickness, a Lorentz term (to model local deviations in bilayer surface curvature), the scale factors for lamellar stacks containing up to five layers, and the scattering length densities of the bilayers and solvent.

The 10% creams were modelled incorporating the paracrystalline lamellar stack parameterisation of Model A1 combined with additional parameters to model the scattering from a separate layer, taken to describe the layer of emulsifier that surrounds each of the oil droplets in the systems; this model is henceforth referred to as Model A2. An additional three parameters were thus modelled in Model A2 accounting for the layer scale factor, thickness and scattering length density.

Model A2 was not used to model fits the SANS profiles for the 4% systems because of the relatively large number of adjustable parameters in this model and because the information contained in these profiles was low. The 4% creams were instead model-fitted combining models that account for scattering from a 1-dimensional para-crystalline stack combined with a power-law to account for the steep rise in Q from 0.01 to 0.02 Å^−1^, Model B (see 3, 12). This model involved a total of 10 parameters: the sample background, the scale factor for the para-crystalline stack, the thickness of the bilayers in the stack, the mean number of bilayers per stack, the *d*-spacing of the bilayers in the stack and the polydispersity on this spacing, the scattering length densities of the bilayers and solvent, and the coefficient and exponent for the Power law used to account for the rise in scattering for *Q* < 0.1 Å^−1^.

Although the SANS measurements on the formulations reported here were made on single preparations (as constrained by the neutron beam time awarded for the experiments), we have shown previously that the results obtained for replicate preparations of the creams are highly reproducible [3]. In the model-fitting of the SANS profiles, the quoted uncertainties on the fitted parameters represent the standard deviations on these estimates obtained from the variance-co-variance matrix in the least-squares optimisation, and where the model-fitting was performed with particular parameters fixed, these parameters are quoted without uncertainties.

## 4. Results

Oil-free ternary systems, prepared with water and emulsifier mixtures of surfactant(s) and co-surfactant(s), have often served—and continue to serve—as structural models for the continuous phases of creams. In the research reported here, we explored systems prepared with emulsifiers comprising SDS combined with one or both of the fatty alcohols hexadecanol, and octadecanol. We sought to investigate the effects of the changes in co-surfactant on the molecular architecture of the lamellar gel network phase formed in these oil-free systems, and in their corresponding cream formulations. We explored the appearance of the cream formulations on the micron scale using bright-field and polarised light microscopy and identified changes in the rheological properties of the creams through shear stress measurements. The changes identified at the micron length scale in the oil-free ternary systems and creams were then related to the convolved structural changes seen in these systems at the nanometre length scale using a combination of SAXS, SANS and WAXS measurements.

### 4.1. Macroscopic Structure

Preparations of 4% and 10% ternary systems containing a 1:1, 1:0 and 0:1 ratio of hexadecanol:octadecanol co-surfactants were shown to have a gel-like appearance and a fluid consistency. The corresponding cream formulations produced smooth, thick and glossy cream-like formulations when the mixtures were cooled during preparation to room temperature. Both the creams and ternary systems appeared homogeneous and free from clumps and precipitants.

### 4.2. Microscopic Structure

**Figure 1:**
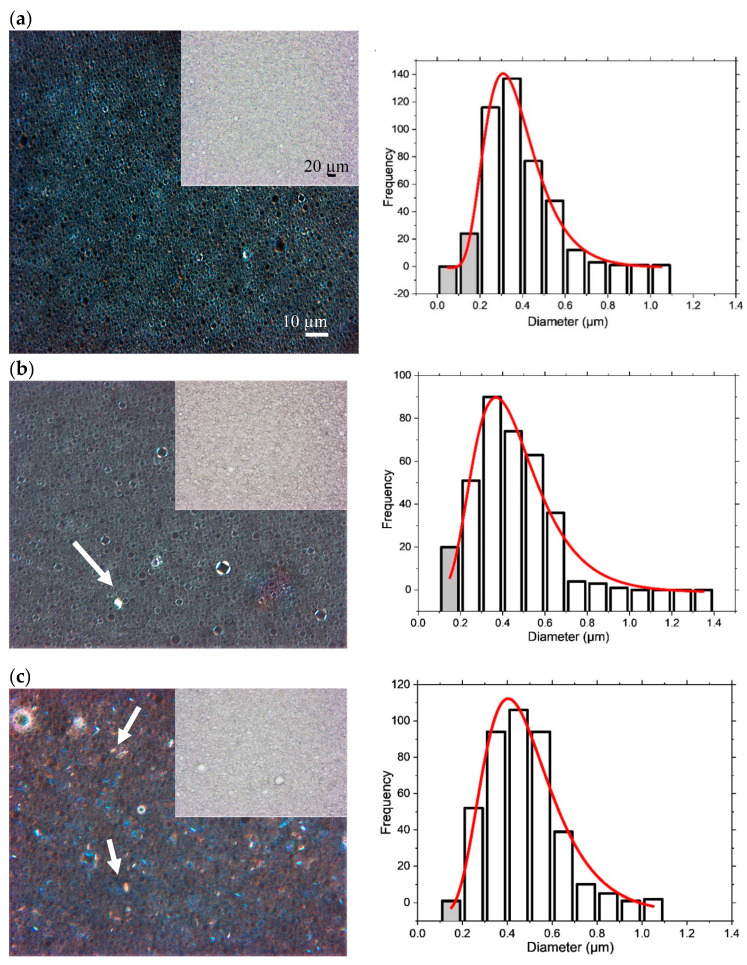
Bright-field and polarised light photomicrographs (left) (at room temperature) of one-day-old 10% creams prepared with a 1:1 (**a**; C4), 1:0 (**b**; C5) or 0:1 (**c**; C6) hexadecanol:octadecanol co-surfactant ratio. Arrows in the polarised light micrographs indicate examples of crystal-like structures. The corresponding oil-droplet diameters measured from the bright-field images are fitted to log-normal distributions (right). The number frequencies of oil droplets with sizes <0.2 microns carry high uncertainties (because the sizes fall below the resolution limit of visible light, 200 nm), and these data are highlighted by shaded bars. Cream compositions are detailed in Methods, Table 1.

Figure 1 shows the micrographs of one-day-old 10% creams obtained under bright-field and polarised light. In the bright-field images, the oil droplets are seen to be dispersed in the continuous aqueous phase. Quantitation of the oil droplet sizes in the 10% creams gave a log-normal size distribution with the mean droplet diameters calculated as ca. 0.29 ± 0.01 µm (comparable to data obtained in [3]), 0.43 ± 0.02 µm and 0.46 ± 0.02 µm for the creams containing mixtures of hexadecanol:octadecanol alcohol co-surfactants as 1:1 (C4), 1:0 (C5) or 0:1 (C6), respectively (Figure 1) and their log standard deviations were determined as 0.35, 0.39 and 0.36, respectively. The slight increase in the mean droplet sizes seen when changing the co-surfactant to include just the hexadecanol or octadecanol, suggests a change in the packing of the monolayer of emulsifier coating the oil droplets. We note too that the droplet diameters obtained for the 4% cream prepared with an equimolar ratio of hexadecanol and octadecanol (Figure S1; Supplementary Material) are significantly larger than those calculated for the corresponding 10% cream (with mean diameters ca. 1.89 ± 0.11 µm vs. 0.28 ± 0.01 µm). As might be expected, therefore, the additional emulsifier present in the 10% creams allows for oil droplets with a smaller surface area to volume ratio.

When the 10% cream formulations are viewed under polarised light, the droplets give rise to birefringence and “Maltese crosses” which are indicative of the presence of a lamellar gel network phase [16]. These are not seen in the 4% creams [3]. It is interesting to note, however, that in addition to the Maltese crosses, there are crystal-like structures that are seen in the polarised light images of the cream formulations containing just hexadecanol (C5; Figure 1b) or octadecanol (C6; Figure 1c) co-surfactant. Whilst in these one-day-old preparations, there are relatively few crystals seen in the cream containing just hexadecanol as co-surfactant, there are large numbers of crystals seen in the micrograph for the cream containing octadecanol (Figure 1c), which suggests that when this latter preparation is cooled, there is excess octadecanol present that does not go to surround the oil droplets and is not incorporated in the bilayers of the gel network, and instead forms non-swollen crystallite structures [25].

In our previous studies, we explored how changes in the amount of total emulsifier (4% vs. 10% *w/w*) in creams influenced the rheology of the formulations. The 4% cream with a 1:1 co-surfactant mixture was found to have a much lower consistency than the corresponding 10% cream [3] and given the absence of Maltese crosses in their polarised light micrographs, it was thus concluded that these systems showed little evidence of a viscoelastic gel network phase.

**Figure 2:**
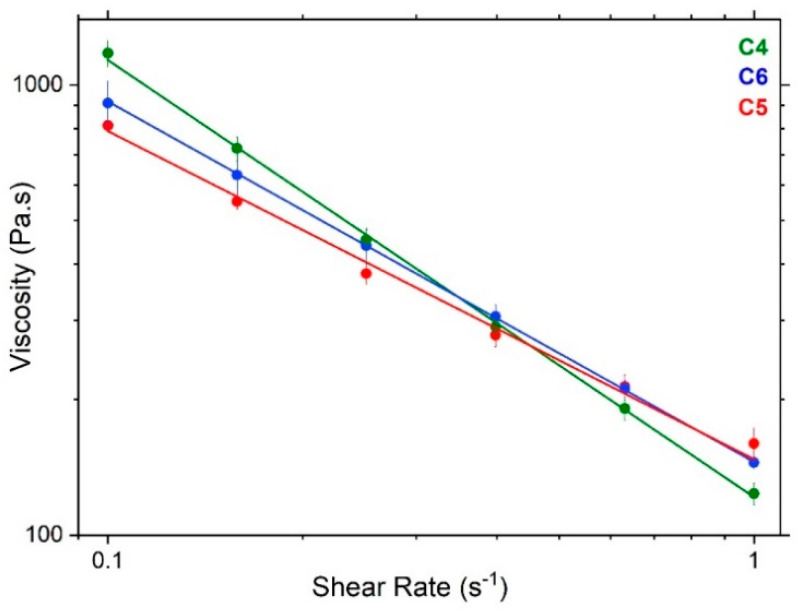
Power-law fitted log-log viscosity flow curves for the 10% creams one day post preparation, prepared with a 1:1 (green; C4), 1:0 (blue; C5) or 0:1 (red; C6) hexadecanol:octadecanol co-surfactant ratio. Power-law model fitted over shear rates 0.1–1 s^−1^, yields consistency (K) indices of 121, 153 and 146 Pa.s^n^ at a shear rate of 1 s^−1^ and flow indices of 0.07, 0.3 and 0.2, respectively. Cream compositions are as provided in Methods, Table 1.

In the current study, we explored the rheological changes that accompany the changes in co-surfactant used in preparing the creams one-day post-preparation. In the log-log profiles of viscosity vs. shear rate (Figure 2), the 10% cream prepared with an equimolar mixture of hexadecanol and octadecanol (1:1; C4) is found to exhibit a viscosity of ca. 1241 ± 131 Pa.s at a shear rate of 0.1 s^−1^, which compares with 812 ± 44 Pa.s and 911 ± 185 Pa.s, respectively, for the formulations containing just hexadecanol (1:0; C5) or octadecanol (0:1; C6). However, we learn that whilst the viscosity of the 1:1 co-surfactant cream (C4) is statistically higher than that for the 1:0 cream (C5) (*p* ≤ 0.05), there is no statistically significant difference found between the viscosities of the 1:1 co-surfactant cream (C4) and that containing just octadecanol as co-surfactant (C6).

All of the cream formulations are found to exhibit shear-thinning behaviour, with flow indices (*n*) of 0.07, 0.3 and 0.2 for the 1:1 (C4), 1:0 (C5) and 0:1 (C6) hexadecanol:octadecanol systems, respectively. The 10% cream prepared with 1:1 hexadecanol:octadecanol (C4), however, seems to show the greatest shear-thinning behaviour, as evidenced by the lower value of its flow index and also by its lower consistency index of ca. 121 Pa.s^n^ at 1 s^−1^, which compares with values of 153 and 146 Pa.s^n^ for the 1:0 and 0:1 co-surfactant creams, respectively (Figure 2). This shear-thinning behaviour is desirable in such cream formulations as it allows them to retain a high viscosity on storage whilst at higher shear rates, they begin to flow so as to allow the cream to be spread easily on the skin.

Rheology measurements for these systems were repeated 1-week post preparation and whilst there were no statistically significant changes in the viscosities of the 1:1 (C4) and 1:0 (C5) co-surfactant creams, there was a significant reduction (*p* < 0.05) seen in the viscosity of the 0:1 octadecanol cream (C6) from 911 ± 185 Pa.s to 860 ± 51 Pa.s at 0.1 s^−1^ for the one-day-old vs. 1-week-old cream. It was not possible, however, to repeat the viscosity measurements on the 1:0 and 0:1 co-surfactant creams at 1-month post preparation since these creams had completely phase-separated by this time. This lack of stability was also noted by Eccleston [11]. For the 1:1 co-surfactant cream, however, there is no significant change in viscosity seen over a 1-month period (data not shown).

### 4.3. Nanoscopic Structure

#### 4.3.1. Small-Angle X-ray Scattering (SAXS)

**Figure 3:**
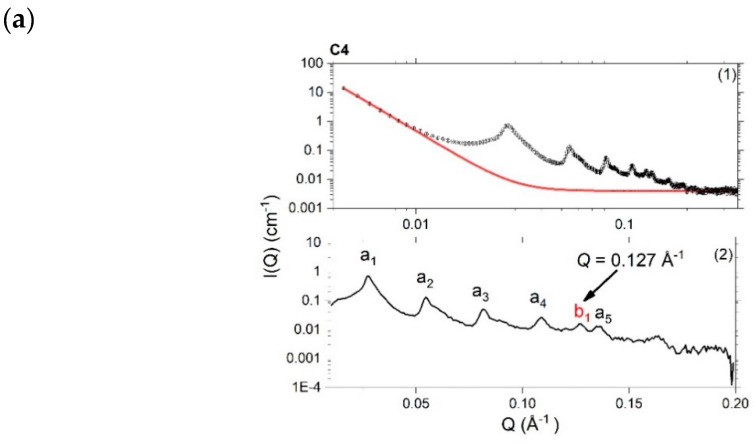

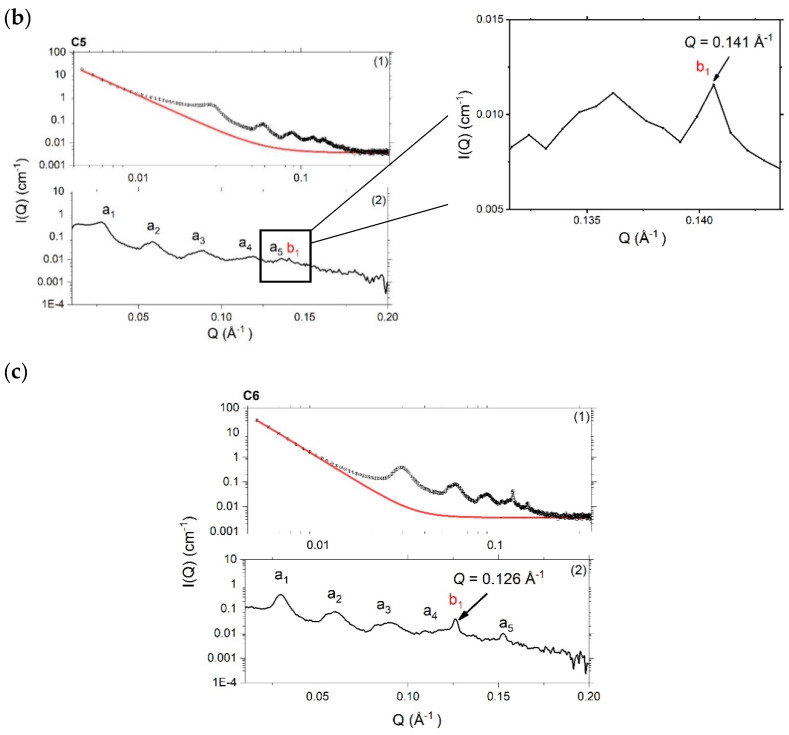
Small-Angle X-ray Scattering (SAXS) profiles recorded at 298 K for 10% creams prepared with a 1:1 (**a**; C4), 1:0 (**b**; C5) or 0:1 (**c**; C6) hexadecanol:octadecanol co-surfactant ratio, with the (*Q*^−4^) Porod scattering model-fitted with a Power law (1; red) and this then subtracted to yield the difference SAXS profile (2). In (2) there are five orders of Bragg reflection (labelled a_1_–a_5_; black) seen for the long periodicity phase and a single reflection (b1; red) seen for a short periodicity phase. These phases have estimated *d*-spacings of ~200 Å and either 50 Å (C4 and C6) or 45 Å (C5), respectively. Inset in (**b**) shows the profile enlarged around the peak arising at *Q* ≈ 0.141 Å^−1^. Error bars on the measured data are subsumed within the plotted symbols. Formulation compositions are as provided in Table 1.

SAXS profiles recorded at 298K of one-day-old 10% creams (Figure 3) and oil-free ternary systems (Figure S2; Supplementary Material) prepared with 1:1, 1:0 or 0:1 ratio of hexadecanol:octadecanol show clear evidence of Bragg peaks, characteristic of the lamellar gel network phase [3]. For the 4% ternary systems and creams, only vestiges of these Bragg peaks are seen (with unclear Bragg reflections beyond the first-order peak) testifying to a much-reduced volume fraction of the gel network bilayers (Figure S3; Supplementary Material). In order to identify the periodicity of the lamellar phase seen in the 10% *w/w* systems, a simple Power law model was used to fit the underlying Q^−4^-dependent (Porod) scattering in the cream samples, and this was subsequently subtracted from the SAXS profiles to reveal the positions of the Bragg peaks more clearly. For the corresponding ternary systems—where there was no such readily identifiable baseline scattering—this tactic could not be employed and so the Bragg peaks were determined from the SAXS profiles directly and the *d*-spacings then computed simply as d = 2π/Q. Bragg reflections were thus identified up to the fifth-order for a lamellar phase with *d*-spacings of approximately 226 Å and 240 Å for the 10% creams and ternary systems, respectively.

In addition to the Bragg peaks associated with this long periodicity lamellar phase (peaks a_1_-a_5_ in Figure 3), some of the SAXS profiles also show an additional peak corresponding to a short periodicity phase (peak b_1_, Figure 3). In the hexadecanol (C5) and octadecanol (C6) 10% cream formulations, these additional peaks are found at *Q* = 0.141 Å^−1^ and 0.126 Å^−1^, respectively, and these correspond to *d*-spacings of 44.6 Å and 49.9 Å. Similar lamellar repeat spacings identified by Kolp and Lutton for these alcohols have been assigned to their sub-alpha polymorphic forms [26], while Valoppi et al. report comparable spacings of 43.6 Å and 48.5 Å, respectively, for 5% blends of hexadecanol and octadecanol with peanut oil [27].

In the 1:1 10% cream (C4), the presence of the additional peak arising at Q = 0.127 Å^−1^ suggests that this cream also contains crystals of octadecanol. However, given the absence of any crystal-like structures in the polarised light images of C4 (vide supra) and the fact that there are no associated peaks seen in the WAXS profile for this cream (vide infra), we deduce that if the formulation truly does contain crystals of octadecanol, the numbers of these must be exceedingly small.

In the corresponding 4% creams, the SAXS profiles for the 1:1 (C1) and 0:1 (C3) systems also show peaks associated with short periodicity phases, but no such peaks are seen in the SAXS profile for the 1:0 (C5) cream, and we note too that the peak for the 1:1 4% cream is shifted to Q = 0.1 Å^−1^.

#### 4.3.2. Small-Angle Neutron Scattering

Complementary SANS experiments were performed on each of the 4% and 10% ternary systems and creams containing protiated components with D_2_O as the solvent. (The use of protiated organic excipients and deuterated solvent here affords a significant improvement in scattering contrast by comparison with the SAXS measurements.) We approach the modelled SANS profiles of the formulations with a varying ratio of the co-surfactant long-chain alcohols in a stepwise manner by first discussing the changes seen in the ternary systems comprising water, SDS and either a 1:1, 1:0 or 0:1 mixture of the hexadecanol:octadecanol co-surfactant(s), and then determining how these fitted parameters are influenced by the addition of liquid paraffin oil in their corresponding creams.

**Figure 4:**
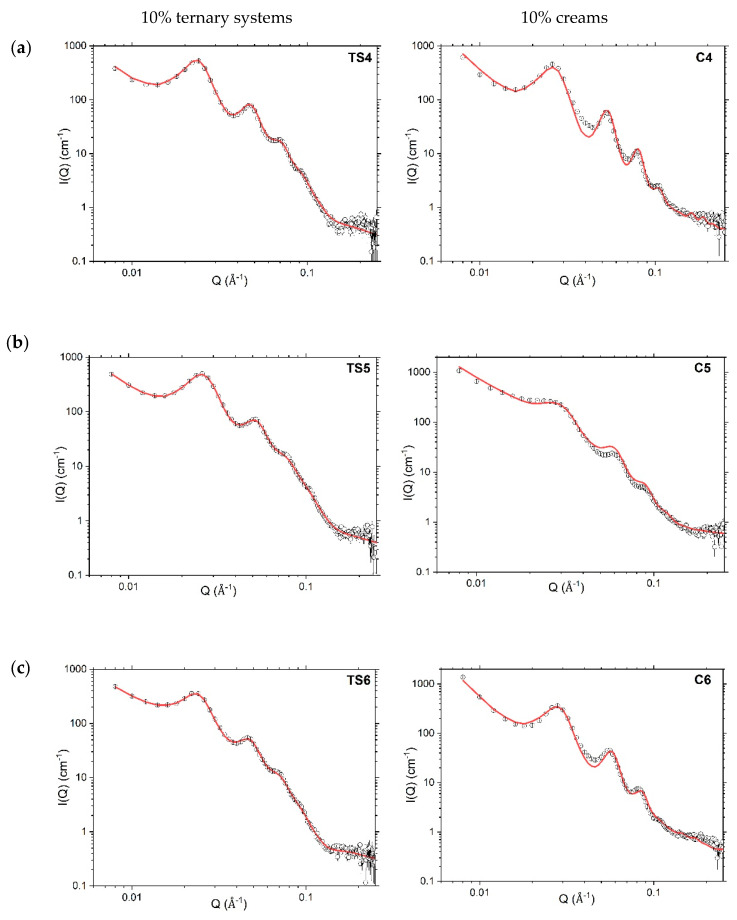
Model-fitted SANS profiles (red lines) recorded at 298 K for 10% ternary systems (TS; left) (Model A1) and creams (**c**; right) (Model A2) prepared with a 1:1 (**a**), 1:0 (**b**) or 0:1 (**c**) ratio of hexadecanol:octadecanol co-surfactants. Error bars on the measured data are subsumed within the plotted symbols. Formulation compositions are provided in Methods, Table 1.

Analytical modelling of the whole SANS profile for the 10% ternary systems (Figure 4) was achieved to a high goodness-of-fit using Model A1 (assuming a para-crystalline lamellar stack). This model is an updated version of a similar model that was used in our previous studies [3,12] and rather than taking the lamellar stacks to involve an identical number of bilayers, the volume fractions of bi-, tri-, tetra- and penta-lamellar stacks were modelled explicitly. With such modelling, we find that single and bi-lamellar stacks account for approximately 20% to 40% of the volume fraction, respectively, with the remaining stacks existing as tri- (~20%), quadri- (~10%) or penta-lamellar (~10%) structures (Table 2). In the corresponding 10% creams—with the exception of the octadecanol cream (C5) where the single bilayers account for ~60% of the volume fraction of the stacks—the fitted volume fractions associated with single bilayers and stacks containing two, three, four and five bilayers follow much the same trend as in the 10% ternary systems (Table 2).

The thicknesses of the bilayers in the 10% ternary systems were model-fitted with thicknesses of 45, 48 and 49 Å, respectively, for the systems containing hexadecanol, octadecanol, and a 1:1 mixture of the two alcohols. These are approximately 3 Å thicker than the bilayers in the corresponding cream formulations (Table 2), and we attribute this increase to small quantities of liquid paraffin oil being incorporated in the gel network bilayers formed in the cream formulations, consistent with our previous findings for 1:1 hexadecanol:octadecanol systems [3,12]. The changes in thickness observed for the bilayers that accompany the changes in the co-surfactant ratio are just as might be expected given the changes in the extended lengths of the alkyl chains of the hexadecanol and octadecanol mixture (C17) and pure hexadecanol (C16) and octadecanol (C18) viz., 46, 44 and 49 Å, respectively [27,28].

The lamellar stacks in the 10% ternary systems were model-fitted with *d*-spacings of 259 ± 26 Å, 232 ± 23 Å and 257 ± 26 Å, respectively for the 1:1, 1:0 or 0:1 hexadecanol:octadecanol co-surfactant systems (Table 2). These spacings are approximately 26, 25 and 41 Å larger than the corresponding fitted *d*-spacings when oil is added to convert the ternary systems to creams. As we noted previously, we attribute this reduction in the lamellar spacing of the creams to a reduced repulsion between the bilayers arising because of structural changes accompanying their incorporation of oil, and the presence within the interlamellar spaces of bicelles formed by SDS mixed with one or both of the co-surfactant long-chain alcohols [3].

In model-fitting the SANS profiles for the creams, there was no attempt made to account for the oblate SDS/co-surfactant ellipsoidal bicelles that we identified in our previous study of creams containing a 1:1 co-surfactant ratio [3]; the small size and low volume fraction of these aggregates within the preparations means that they make negligible contributions to the scattering arising from samples prepared with protiated materials dispersed in D_2_O.

The SANS profiles for the 10% creams were modelled by combining Model A1—used to fit the 10% ternary systems—with parameters that account for the thickness of the surfactant/co-surfactant monolayer surrounding the oil droplets (Figure 4). Thus, whereas in our previous studies we used a Power law model to fit the SANS profiles of the creams below Q < 0.01 Å^−1^ and took the Power law exponent to describe the diffuse surfactant/co-surfactant layer coating the oil-droplets [3,12], in this study we determine the thickness of this layer as 26, 24 and 27 Å (Table 3) in the 1:1, 1:0 and 0:1 hexadecanol:octadecanol 10% creams, respectively. The differences seen are consistent with the variations in the calculated extended lengths of their co-surfactant alkyl chains.

**Figure 5:**
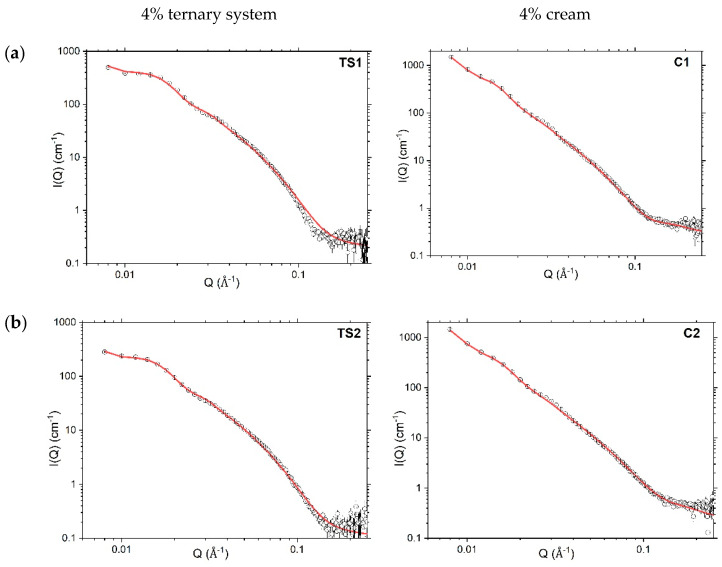

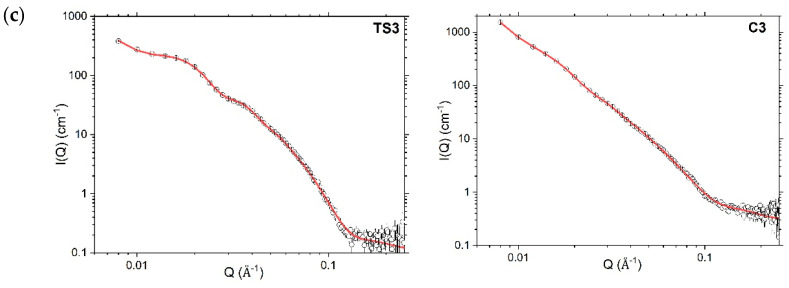
Model-fitted SANS profiles (red lines) recorded at 298 K for 4% ternary systems (TS) (left; Model A1) and creams (C) (right; Model B) prepared with a 1:1 (**a**), 1:0 (**b**) or 0:1 (**c**) ratio of hexadecanol:octadecanol co-surfactants. Error bars on the measured data are subsumed within the plotted symbols. Formulation compositions are provided in Methods, Table 1.

Whilst the SANS profiles recorded for the 10% systems show well-defined Bragg peaks (Figure 4), the 4% ternary systems and creams have rather low information content with only vestiges of these Bragg peaks seen (Figure 5). The 4% ternary systems were modelled using Model A1 as in their corresponding 10% systems. With such modelling, we learn that the 4% ternary systems contain ca. 55% single bilayers by volume and 45% by volume of stacks with just two bilayers, and that the weighted mean numbers of bilayers per stack for the 4% *w/w* (Table 3) and 10% systems (Table 2), are ca. 1.4 and 2.5 respectively.

Although the bilayers in the 4% ternary systems were model-fitted with thicknesses that are generally lower by comparison with the 10% ternary systems (Table 2 vs. Table 3) their thicknesses come with high uncertainties and fits of comparable quality were obtained if the thicknesses were fixed with values of 48, 45, and 49 Å as found for the 10% ternary systems with a 1:1 (TS4), 1:0 (TS5) or 0:1 (TS6) hexadecanol:octadecanol ratio, respectively.

Given the rather low information content of the 4% creams and the fact that these formulations also contain oil, a more parsimonious approach in modelling was required. These creams were modelled using Model B (Figure 5) (the same model used in our previous SANS modelling of aqueous creams [3])—assuming scattering from a para-crystalline lamellar stack (with all stacks having the same lamellarity) combined with a simple Power law model to account for the scattering at Q values below 0.02 Å^−1^. The Power law exponent −3 indicating a mass fractal dimension was used to describe the nature of the monolayer surrounding the oil droplets [27,29]. in addition, in keeping with our observations of the model-fitted bilayer thicknesses of the 4% vs. 10% ternary systems remaining the same, the model-fitting for the 4% creams was carried out with the bilayer thicknesses kept the same as in the 10% *w/w* creams and with no fitted polydispersity (Table 3).

Fitted SANS profiles of both the 4% ternary systems and creams (Table 3) gave rise to a d-spacing that is around 100 Å larger (in some cases around 150 Å larger) than that for the corresponding 10% systems (Table 2). Since in both the ternary systems and creams, the thickness of the bilayers in the lamellar stack remains unchanged when the emulsifier concentration is increased from 4% *w/w* to 10% *w/w* (Table 2 vs. Table 3, respectively), the changes seen in d-spacing thus arise because of a decrease in thickness of the interlamellar water region.

#### 4.3.3. Variations in SANS Profiles with Temperature

**Figure 6:**
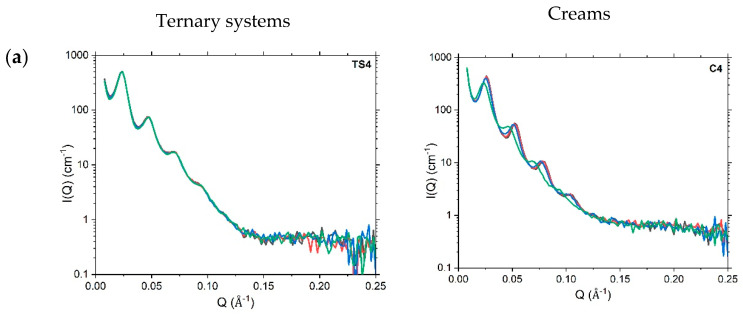

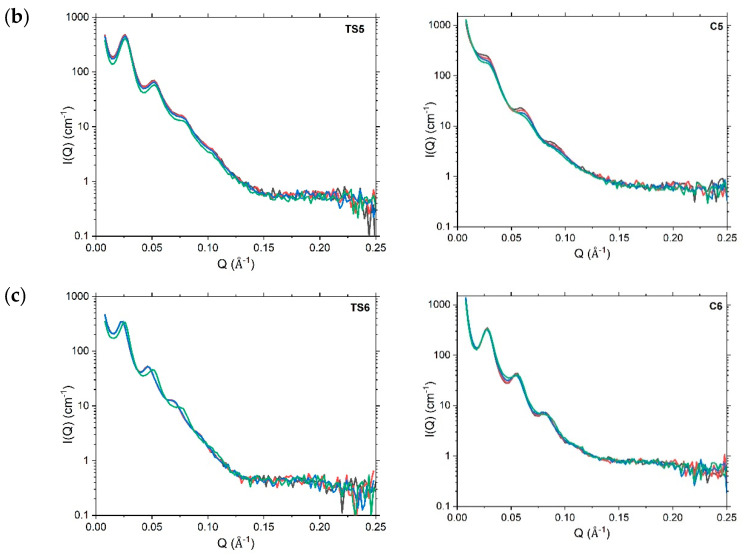
SANS profiles (model fits not shown) obtained at 298 (black), 305 (red), 310 (blue) and 318 (green) K (±0.1 K) for 10% *w/w* ternary systems (left) and emulsifier creams (right) and prepared with a 1:1 (**a**), 1:0 (**b**) or 0:1 (**c**) ratio of hexadecanol:octadecanol co-surfactants. Error bars on the measured data are subsumed within the plotted symbols. Formulation compositions are provided in Methods, Table 1.

SANS measurements were performed on the 4% *w/w* (Figure S4, Supplementary Material) and 10% *w/w* (Figure 6) ternary systems and creams to identify how a change in temperature would perturb the nanostructures of these systems and their gel network phases. The measurement temperatures of 298, 305, 310, and 318 K, were used to simulate room, skin, body and high storage temperatures, respectively.

With no significant changes visible in the SANS profiles for the 4% ternary systems and creams, we conclude that these systems are unaffected by a change in temperature (Figure S4, Supplementary Material). For the 10% *w/w* ternary systems, the SANS profiles recorded at 318 K were modelled (using Model 1A) as shown in Figure S5 and the fitted parameters show no change in the nanostructure—*d*-spacings or bilayer thicknesses—in the 1:1 and 1:0 hexadecanol:octadecanol co-surfactant systems. However, for the pure octadecanol system (0:1; TS6), we see a reduction in the gel network *d*-spacing from 257 Å to 240 Å when increasing the temperature from 298 K to 318 K (Table 4). We saw the same reduction in *d*-spacing when we performed SANS measurements on an oil-free lipid barrier formulation (unpublished data) [30], indicating that some of the inter-lamellar water transfers out from the gel network into the bulk continuous phase. When these formulations were returned to room temperature, however, there was a shift in the Bragg peaks back to their original positions suggesting that the water readily transfers back from the bulk into the gel network phase.

Changes in the combined volume fraction of the gel network lamellar stacks caused by a change in temperature can be judged simply by comparing the intensities of the Bragg peaks in the respective SANS profiles. By such means, we note that the 1:1 10% ternary system is unaffected by the increase in temperature and that for the other two (single fatty alcohol) systems, the rise in temperature leads to a decrease in the volume fraction of lamellar stacks, and this is more pronounced for the 0:1 (octadecanol; TS6) system than for the 1:0 (hexadecanol; TS5) system. For all ternary systems, the increase in temperature seems to lead to a small reduction in the proportion of single bilayers, and in the 1:0 and 1:1 systems, this is mirrored by small increases in the proportions of bi- and tri-lamellar stacks and in the 0:1 system an increase in the proportion of the penta-lamellar stacks (Table 2 vs. Table 4).

The corresponding 10% creams (Figure 6) show rather different behaviour when the temperature is increased to 318 K. There are only small changes seen in the positions of Bragg peaks for the 1:0 and 0:1 systems that lead to a small reduction in their d-spacings (Table 4). The Bragg peaks seen in the profile for the 1:1 co-surfactant cream (C4), however, clearly shift to lower Q (Figure 6). The elevation of temperature thus leads to an increase in the *d*-spacing of the gel network from 233 Å to 253 Å (Table 2 vs. Table 4), and we conclude, therefore, that there is a transfer of water into the inter-lamellar region. Niemi and Laine [31] and Rowe and Bray [32] have previously proposed that three distinct water phases exist within oil-in-water creams. In addition to the bulk (free) water phase, it is suggested that there is water confined within the interlamellar spaces of the gel network, and water that is closely associated with the liquid crystalline network surrounding the oil droplets. The changes in d-spacing that we see here most likely arise as a result of water moving between the inter-lamellar fixed water and the bulk water [32].

Since the SANS profiles for the heated creams return to overlay their original positions when the formulations are cooled (data not shown)—with the same observations made on lipid barrier formulations as previously investigated (unpublished data [30])—we assume that the water that is lost or gained from the gel network does not completely leave the system. For the cream containing a 1:1 co-surfactant ratio (C4) in storage, therefore, we would expect that as long as it is kept within a closed container, any transient increase in temperature (up to 318 K) is unlikely to compromise its nanostructure significantly.

In addition to these changes in d-spacing, we also see a progressive reduction in the volume fraction (indicated by reduced intensities of the Bragg peaks) of the gel network as the temperature is increased for the hexadecanol surfactant cream (C5) (Figure 6); no such change is seen in the octadecanol cream (C6) and a reduction in the volume fraction of the gel network is only seen at 318 K for the 1:1 co-surfactant cream (C4).

#### 4.3.4. Wide-Angle X-ray Scattering (WAXS)

**Figure 7:**
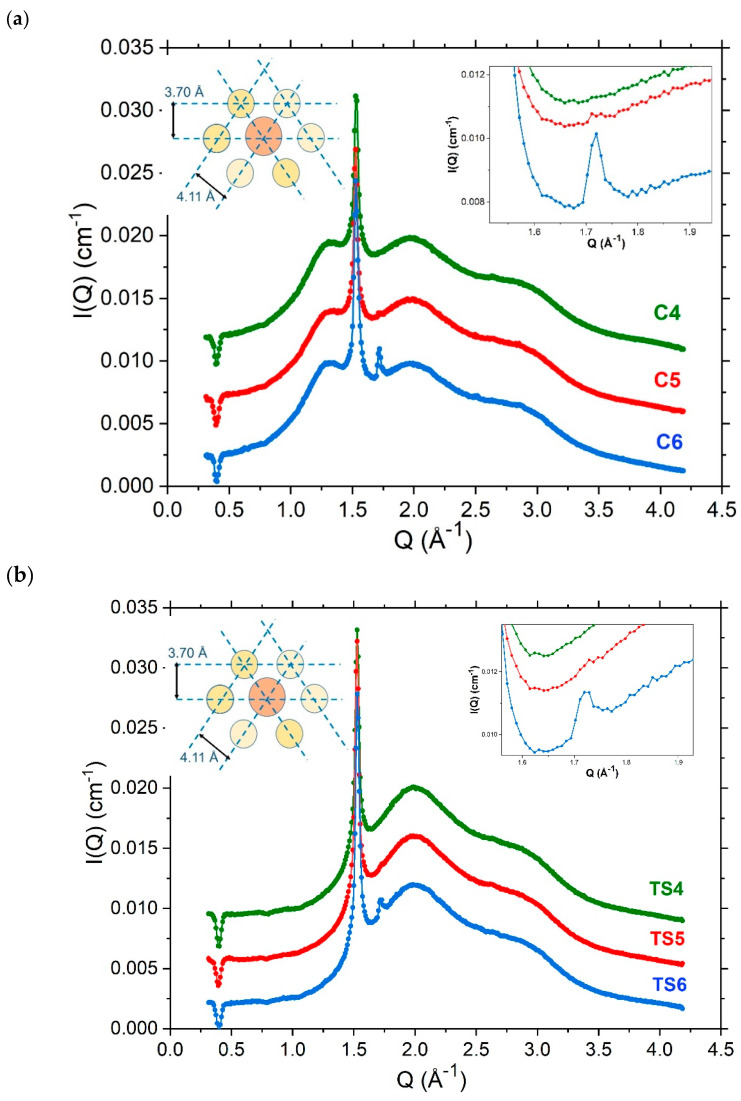
Wide-Angle X-ray Scattering (WAXS) profiles (offset on the ordinate for clarity) recorded at 298 K for 10% creams (**a**) and ternary systems (**b**) containing a 1:1 (green; C4, TS4), 1:0 (red; C5, Table 2. or 0:1 (blue; C6, TS6) hexadecanol:octadecanol co-surfactant ratio. Insets in (**a**) and (**b**) show the profiles enlarged around the peak arising at *Q* ≈ 1.7 Å^−1^ corresponding to an orthorhombic packing of the co-surfactant(s) with the additional schematics depicting the packing of the co-surfactant alcohols and SDS in the layer surrounding each oil droplet. Formulation compositions as provided in Methods, Table 1.

WAXS measurements performed on these formulations provide insight as to the lateral organisation of the molecules in their respective aggregates. The profiles recorded at 298 K for one-day-old preparations of the 4% *w/w* (Figure S6, Supplementary Material) and 10% *w/w* (Figure 7) emulsifier creams and their corresponding ternary systems all give rise to a sharp reflection at 4.11 Å. This is characteristic of the α-crystalline hexagonal packing of the hydrocarbon chains in gel phase bilayers present when the systems are cooled to temperatures below the phase transition temperatures of the component amphiphiles [3,16]. Additionally, it is evident that the shoulder present at a 4.65 Å spacing in the 4% *w/w* and 10% creams (Figure 7a and Figure S6a, Supplementary Material) is absent from the profiles of their corresponding oil-free ternary systems (Figure 7b and Figure S6b, Supplementary Material). We thus attribute this 4.65 Å spacing to a co-existing liquid crystalline phase which we take to arise from a loosely packed monolayer of SDS and one or both of the long-chain alcohol co-surfactant(s) that surround the oil droplets [3].

When the formulations were prepared with a single fatty alcohol as co-surfactant, there was an additional small but sharp peak seen for the 10% *w/w* cream and ternary system (Figure 7) and the 4% *w/w* cream (Figure S6a, Supplementary Material) prepared with octadecanol; and remnants of this peak were seen in the WAXS profile for the 10% *w/w* cream containing hexadecanol (Figure 7). Octadecanol, therefore, seems to have a higher propensity to form crystallites than hexadecanol given the relative intensities of the peaks seen in their respective WAXS profiles, and the fact that octadecanol crystallites were visibly abundant in the polarised light micrographs for the C6 cream, and the corresponding Bragg peaks identified in the SAXS profiles for the octadecanol 4% (C3) and 10% *w/w* (C6) emulsifier creams (vide supra).

**Figure 8:**
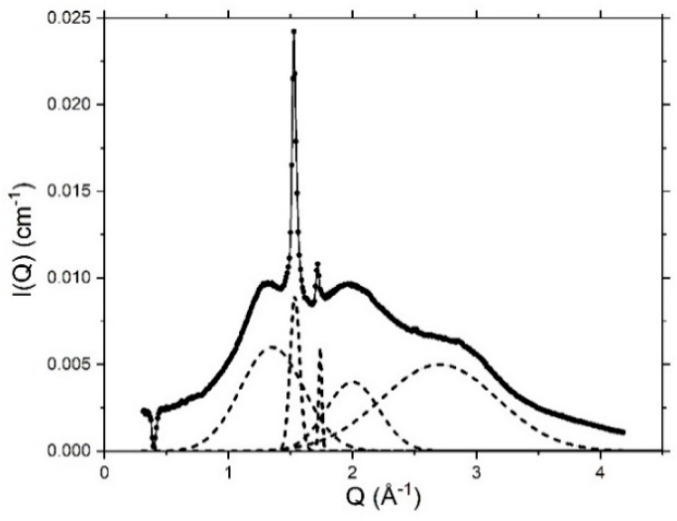
WAXS profile (solid line) measured at 298 K for a 10% cream prepared with octadecanol (C6) as co-surfactant and the deconvolution of the profile (dotted line) revealing peaks corresponding to lateral spacings (left to right) of 4.65 Å, 4.11 Å, 3.70 Å, 3.12 Å and 2.36 Å. Formulation composition is provided in Methods, Table 1.

Deconvolution of the WAXS profile for these systems—as shown in Figure 8 for the 10% cream prepared with octadecanol (C6)—reveals a 3.70 Å *d*-spacing for this (additional) peak which we assign to the orthorhombic packing of the octadecanol and hexadecanol crystallites [33]. Valoppi et al. [27] also reported an orthorhombic arrangement of the hexadecanol and octadecanol present in 5% binary systems where the co-surfactants were mixed with peanut oil, and studies by Karl et al. found that these alcohols can exist in different polymorphic forms, with the major, most stable form existing at lower temperatures being the orthorhombic form [34]. In their study, Karl et al. also associate the short spacing of 43.98 Å that was identified in the SAXS profile for hexadecanol to an orthorhombic packing and this is consistent too with the observations made in this study. Whereas in the hexagonal state, the hydrocarbon chains of the fatty alcohols can freely rotate around their long axes, in the orthorhombic state there is less lateral movement as the molecules exist in a solid state and are packed closely in one direction [35].

Through the WAXS profiles obtained for the 10% *w/w* systems, we learn that the high concentration of emulsifier gives rise to some crystallites in the ternary systems and—given the intensity of the peak—these crystallites appear to be more abundant in the cream formulations due to the solubilisation of the fatty alcohols with liquid paraffin oil. The 4% systems have a reduced concentration of emulsifier and only the cream formulation (Figure S6a, Supplementary Material) shows an orthorhombic packing in the octadecanol cream (C6) possibly as a consequence of the octadecanol crystallites solubilised with oil.

The additional broad peaks in the de-convoluted WAXS profiles for the 4% *w/w* and 10% *w/w* creams and ternary systems (as shown in Figure 8) revealing *d*-spacings of 2.36 Å and 3.12 Å, which are attributed to the interatomic distributions of O–O, O–H and H–H for water in these systems [3].

## 5. Conclusions

Topical formulations are generally formulated with a view that their internal lamellar gel network structure will act to mimic the natural lipid arrangement of the stratum corneum of the skin and thus repair the compromised barrier function. The studies presented here were focused on creams based around Aqueous Cream B.P [36]) and although the surfactant in the formulation, SDS, can cause skin irritancy, the other components of the formulation, such as liquid paraffin oil and hexadecanol and octadecanol (either as pure alcohols or as a commercially available mixture) still form the basis of many products that are used in the dermatology field to treat dry skin conditions. Whilst in our previous studies we elucidated the microstructure of creams based on Aqueous Cream B.P and explored how the structure is influenced by the presence of an antimicrobial additive and variations in the nature of the surfactant used [3,12], in the studies reported here, we highlight the importance of the fatty alcohol co-surfactant mixture and report the implications of changes in co-surfactant on the gel network structure and on the consequences of this as regards their thermotropic behaviour and temporal stability.

Lamellar gel network formation is evidenced in both the oil-free ternary systems and creams containing 10% *w/w* emulsifier, with significant volume fractions of tri-, quadri- and penta-lamellar stacks of co-surfactant bilayers. In the 4% *w/w* emulsifier systems, however, we see only single bilayers and bi-lamellar stacks. The inter-lamellar spacings and bilayer thicknesses change when the ternary systems are converted to creams and these changes are consistent with the incorporation of oil in the bilayers in the creams.

When the ratio of the co-surfactants was varied, the changes seen in bilayer thicknesses in the ternary systems and creams as well as the thicknesses of the monolayers surrounding the oil droplets in the creams mirror the changes in the lengths of the alkyl chains of the added co-surfactants. The consequence of the co-surfactant-dependent changes in the structure of the creams—and in particular, the changing nature of the monolayers surrounding the oil droplets—are seen to lead to macroscopic changes such that the mean oil droplet sizes in the creams containing only one of the fatty alcohol co-surfactants are significantly larger than that in the cream containing both alcohols.

Crystal-like structures were also identified in the single alcohol creams with these present in their orthorhombic crystalline forms. The changes in the mean oil droplet sizes and/or the presence of these co-surfactant crystals seem to adversely affect the stability of the single co-surfactant creams, such that the octadecanol cream shows a significant reduction in viscosity just 1-week post preparation, and both of the single co-surfactant creams completely phase separating within 1-month of storage.

As regards thermal stability, we find that the ternary systems show very little change in structure for increases in temperature up to 318 K, but with the temperature raised to 318 K there are clear structural changes seen in the ternary systems and rather different patterns of change in the structures of their partner cream formulations. These changes—which are reversed on cooling—are attributed to the movement of water between the inter-lamellar spaces and the bulk.

Through the combination of findings presented here, we discover that creams containing a mix of co-surfactants afford improved stability and better sensory properties than those containing a single co-surfactant species. We learn too that there are differences in lamellar structure that exist between the ternary systems and creams, and also differences in the behaviour and thus in the structures of the formulations when they are subject to elevated temperatures, demonstrating that the ternary systems are imperfect models of the continuous phase of creams.

## Figures and Tables

**Table 1:** Chemical composition of aqueous creams (C1–C6) ^a^.

Formulations	Total Emulsifier Concentration	Hexadecanol	Octadecanol	SDS	Liquid Paraffin	Water ^b^
	Percentage % (*w/w*)					
C1	4	1.70	1.90	0.40	20	76
C2	4	3.60	--	0.40	20	76
C3	4	--	3.60	0.40	20	76
C4	10	4.25	4.75	1.00	20	70
C5	10	9	--	1.00	20	70
C6	10	--	9	1.00	20	70

^a^ The corresponding oil-free “ternary systems” (abbreviated as TS1, TS2, etc.) were prepared by adjusting the level of water in the formulation as 96% and 90% *w/w*, for the 4% and 10% ternary systems, respectively. ^b^ Formulations were prepared in an equivalent volume of D_2_O when prepared for neutron scattering experiments.

**Table 2:** Model fitted parameters for the Small-Angle Neutron Scattering (SANS) profiles at 298 K of ternary systems (TS4–TS6) ^a^ and aqueous creams (C4–C6) and containing 10% *w/w* emulsifier concentration and either a 1:1, 1:0 or 0:1 ratio of hexadecanol:octadecanol co-surfactants.

	(1:0)		(1:1)		(0:1)	
	TS5	C5	TS4	C4	TS6	C6
Bilayer thickness (Å) ^b^	45 ± 1	47	48 ± 1	50	49 ± 1	52
Polydispersity on bilayer thickness	0.2	0.2	0.2	0.2	0.2	0.2
*d*-spacing (Å)	232 ± 1	207 ± 1	259 ± 1	233 ± 1	257 ± 1	216 ± 1
Polydispersity on *d*-spacing	0.1	0.08	0.1	0.06	0.1	0.09
Lorentz term	95 ± 1	209 ± 2	108 ± 1	127 ± 6	133 ± 2	381 ± 7
Unilamellar	0.20	0.57	0.20	0.23	0.35	0.16
Bilamellar	0.40	0.28	0.38	0.38	0.32	0.40
Trilamellar	0.22	0.15	0.19	0.19	0.17	0.25
Quadrilamellar	0.10	0	0.10	0.08	0.10	0.09
Pentalamellar	0.08	0	0.13	0.12	0.06	0.10
Oil droplet layer thickness (Å)	--	24 ± 2	--	26 ± 0.5	--	27 ± 0.3

^a^ Ternary systems and creams were modelled using Model A1 and A2, respectively. ^b^ No uncertainties shown on the quoted bilayer thicknesses for C4, C5 and C6 as these values were fixed.

**Table 3:** Model fitted parameters for the SANS profiles at 298 K of ternary systems (TS1–TS3) ^a^ and aqueous creams (C1–C3) containing 4% *w/w* emulsifier concentration and a 1:1, 1:0 or 0:1 ratio of hexadecanol:octadecanol co-surfactants.

	(1:0)		(1:1)		(0:1)	
	TS2	C2	TS1	C1	TS3	C3
Bilayer thickness (Å) ^b^	41 ± 6	47	44 ± 4	50	49 ± 6	52
Polydispersity on bilayer thickness	0.15	--	0.11	--	0.14	--
*d*-spacing (Å) ^c^	374	383	357	400	328	365
Polydispersity on *d*-spacing	0.2	0.3	0.2	0.2	0.1	0.2
Lorentz term	230 ± 3	--	--	298 ± 9	--	245 ± 4
Unilamellar	0.59	--	0.54	--	0.54	--
Bilamellar	0.41	--	0.46	--	0.46	--
Trilamellar	0	--	0	--	0	--
Quadrilamellar	0	--	0	--	0	--
Pentalamellar	0	--	0	--	0	--
Power law exponent	--	3.4	--	3.1	--	3.3

^a^ Ternary systems and creams were modelled using Models A1 and B, respectively. ^b^ No uncertainties are shown on the quoted bilayer thicknesses for C4, C5 and C6 as these values were fixed. ^c^ the reported *d*-spacings for the ternary systems (TS1–TS3) and creams (C1–C3) are presented without uncertainties on the fitted values given their large fitted polydispersity values.

**Table 4:** Model fitted parameters for the SANS profiles at 318 K of ternary systems (TS4–TS6) ^a^ and creams (C4–C6) containing 10% *w/w* emulsifier concentration and either a 1:1, 1:0 or 0:1 ratio of hexadecanol:octadecanol co-surfactants.

	(1:0)		(1:1)		(0:1)	
	TS5	C5	TS4	C4	TS6	C6
Bilayer thickness (Å)^b^	45 ± 1	47	48 ± 1	50	49 ± 1	52
Polydispersity on bilayer thickness	0.2	0.2	0.2	0.2	0.2	0.2
d-spacing (Å)	231 ± 1	198 ± 1	258 ± 1	253 ± 1	240 ± 1	212 ± 1
Polydispersity on d-spacing	0.1	0.10	0.1	0.08	0.1	0.08
Lorentz term	95 ± 1	344 ± 6	109 ± 1	117 ± 7	98 ± 1	379 ± 8
Unilamellar	0.16	0.63	0.17	0.38	0.34	0.0
Bilamellar	0.42	0.2	0.4	0.43	0.3	0.52
Trilamellar	0.24	0.17	0.2	0.09	0.13	0.32
Quadrilamellar	0.09	0	0.11	0.10	0.1	0.11
Pentalamellar	0.09	0	0.12	0.0	0.13	0.05

^a^ Ternary systems and creams were modelled using Models A1 and A2, respectively. ^b^ No uncertainties are shown on the quoted bilayer thicknesses for C4, C5 and C6 as these values were fixed.

## Supplementary Materials

The following are available online at https://www.mdpi.com/1999-4923/12/9/864/s1, Figure S1: Log-normal distribution showing oil-droplet diameters obtained from the bright-field images of 4% creams prepared with an equimolar ratio hexadecanol:octadecanol. Formulation composition given in Methods, Table 1, Figure S2: SAXS profiles (offset on the ordinate for clarity) recorded at room temperature for 10% ternary systems prepared with a 1:1 (green; TS4), 1:0 (red; TS5) or 0:1 (blue; TS6) hexadecanol:octadecanol co-surfactant ratio. Error bars on the measured data are subsumed within the plotted symbols. Formulation compositions are given in Methods, Table 1, Figure S3: SAXS profiles (offset on the ordinate for clarity) recorded at room temperature for 4% ternary systems (a; TS1–TS3) and creams (b; C1–C3) prepared with a 1:1 (green), 1:0 (red) or 0:1 (blue) hexadecanol:octadecanol co-surfactant ratio. Error bars on the measured data are subsumed within the plotted symbols. Inset in (b) shows enlarged SAXS profiles, highlighting position of the peaks in C1 and C3 at *Q* ≈ 0.1 Å^−1^ and *Q* = 0.126 Å^−1^, respectively. Formulation compositions are given in Methods, Table 1, Figure S4: SANS profiles (model fits not shown) obtained at 298 (black), 305 (red), 310 (blue) and 318 (green) K (± 0.1 K) for 4% ternary systems (left; TS1–TS3) and emulsifier creams (right; C1–C3) prepared with a 1:1 (a), 1:0 (b) or 0:1 (c) ratio of hexadecanol:octadecanol co-surfactants. Error bars on the measured data are subsumed within the plotted symbols. Formulation compositions are given in Methods, Table 1, Figure S5: Model-fitted SANS (red line; Model A1) profiles recorded at 318 K for 10% ternary systems prepared with a 1:1 (a; C4), 1:0 (b; C5) or 0:1 (c; C6) hexadecanol:octadecanol co-surfactant ratio. Fitted parameters gave rise to a *d*-spacing of 258, 231 and 240 Å and bilayer thicknesses 48 Å, 45 Å and 49 Å for TS4 (a), TS5 (b) and TS6 (c), respectively. Error bars on the measured data are subsumed within the plotted symbols. Formulation compositions are given in Methods, Table 1, Figure S5: WAXS profiles (offset on the ordinate for clarity) of 4% emulsifier creams (a) and ternary systems (b) prepared with a 1:1 (green; C1), 1:0 (red; C2) or 0:1 (blue; C3) hexadecanol:octadecanol co-surfactant ratio. Formulation compositions are given in Methods, Table 1.
