# Supplementary material for: The Influence of Co-Surfactants on Lamellar Liquid Crystal Structures Formed in Creams
Source: Pharmaceutics. 2020 Sep 11;12(9):864. doi: 10.3390/pharmaceutics12090864 (PMC7557764; doi:10.3390/pharmaceutics12090864)
Supplement: Supplementary file 1 [file pharmaceutics-12-00864-s001.pdf]

pharmaceutics

Supplementary Materials: The Influence of Co-Surfactants on Lamellar Liquid Crystal Structures Formed in Creams

Delaram Ahmadi, Najet Mahmoudi, Richard K. Heenan, David J. Barlow and M. Jayne Lawrence

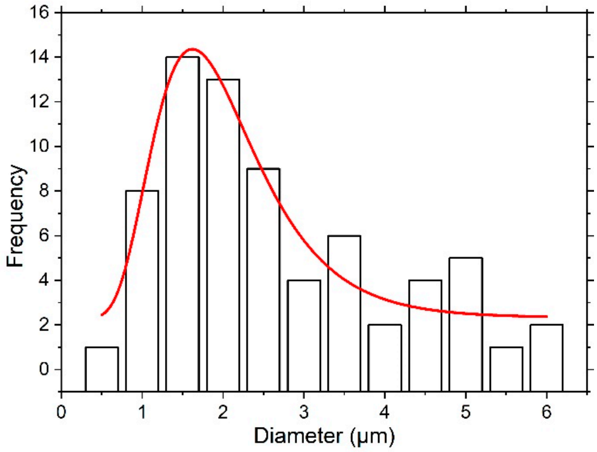

Figure S1. Log-normal distribution showing oil-droplet diameters obtained from the bright-field images of 4% creams prepared with an equimolar ratio hexadecanol:octadecanol. Formulation composition given in Methods, Table 1.

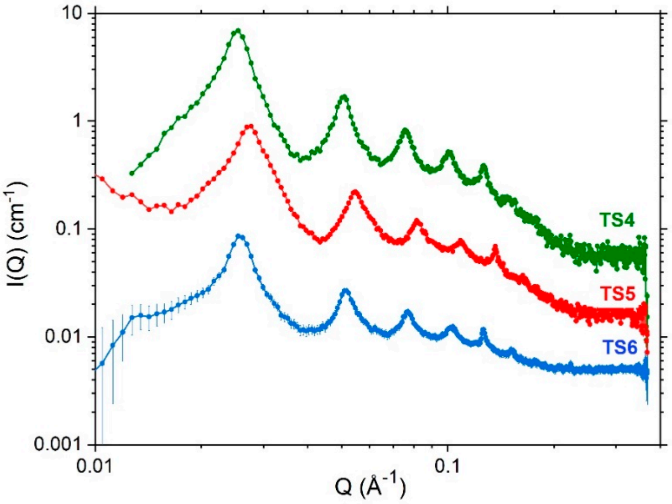

Figure S2. SAXS profiles (offset on the ordinate for clarity) recorded at room temperature for 10% ternary systems prepared with a 1:1 (green; TS4), 1:0 (red; TS5) or 0:1 (blue; TS6) hexadecanol:octadecanol co-surfactant ratio. Error bars on the measured data are subsumed within the plotted symbols. Formulation compositions are given in Methods, Table 1.

pharmaceutics

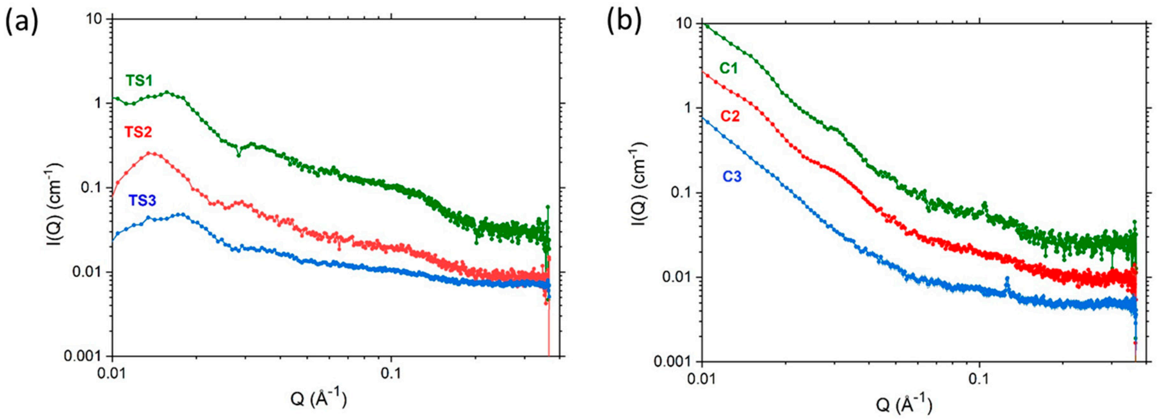

Figure S3. SAXS profiles (offset on the ordinate for clarity) recorded at room temperature for 4% ternary systems (**a**; TS1–TS3) and creams (**b**; C1–C3) prepared with a 1:1 (green), 1:0 (red) or 0:1 (blue) hexadecanol:octadecanol co-surfactant ratio. Error bars on the measured data are subsumed within the plotted symbols. Inset in (b) shows enlarged SAXS profiles, highlighting position of the peaks in C1 and C3 at $Q \approx 0.1$ Å^{-1} and $Q = 0.126$ Å^{-1}, respectively. Formulation compositions are given in Methods, Table 1.

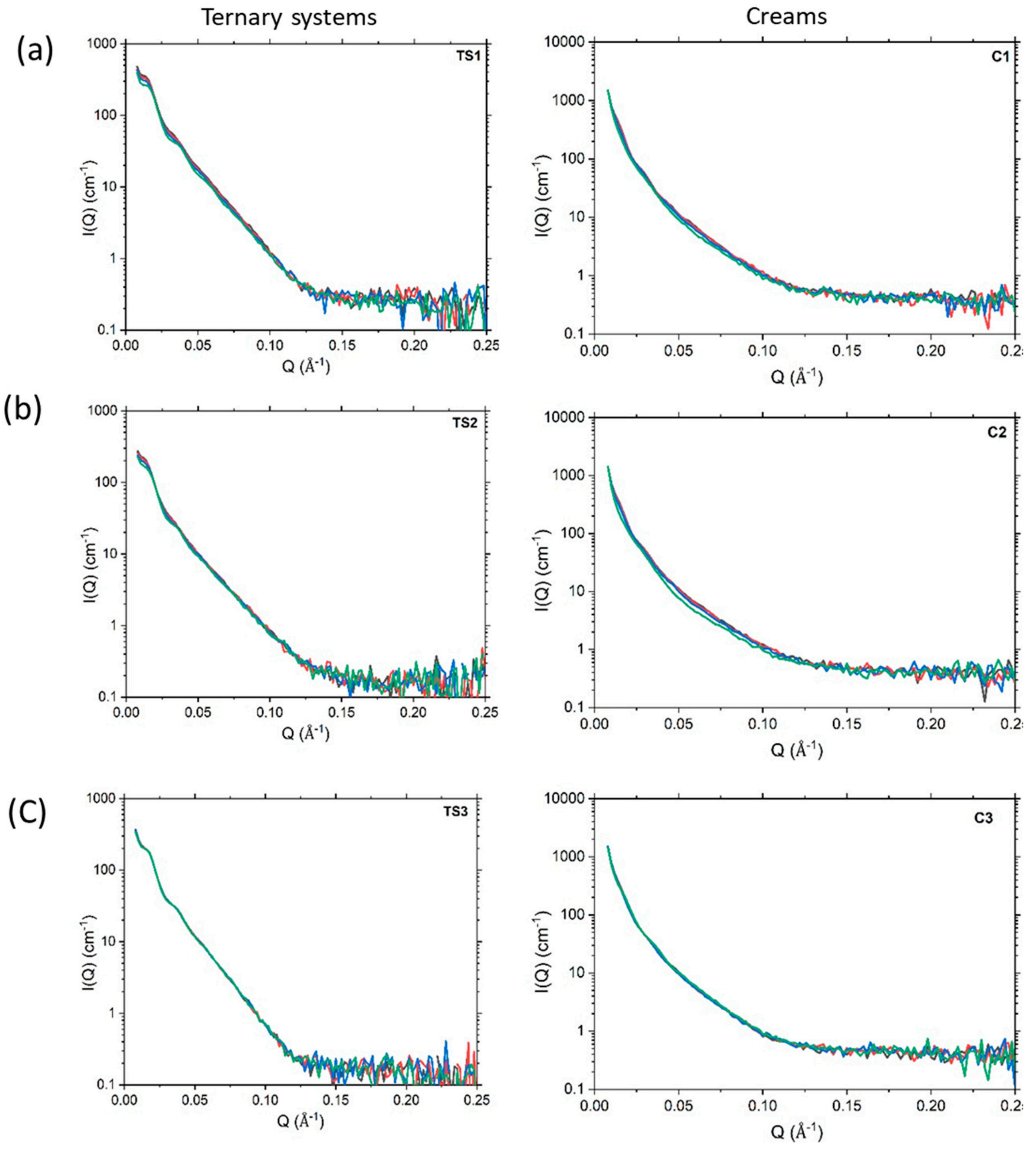

Figure S4. SANS profiles (model fits not shown) obtained at 298 (black), 305 (red), 310 (blue) and 318 (green) K (± 0.1 K) for 4% ternary systems (left; TS1–TS3) and emulsifier creams (right; C1–C3) prepared with a 1:1 (**a**), 1:0 (**b**) or 0:1 (**c**) ratio of hexadecanol:octadecanol co-surfactants. Error bars on the measured data are subsumed within the plotted symbols. Formulation compositions are given in Methods, Table 1.

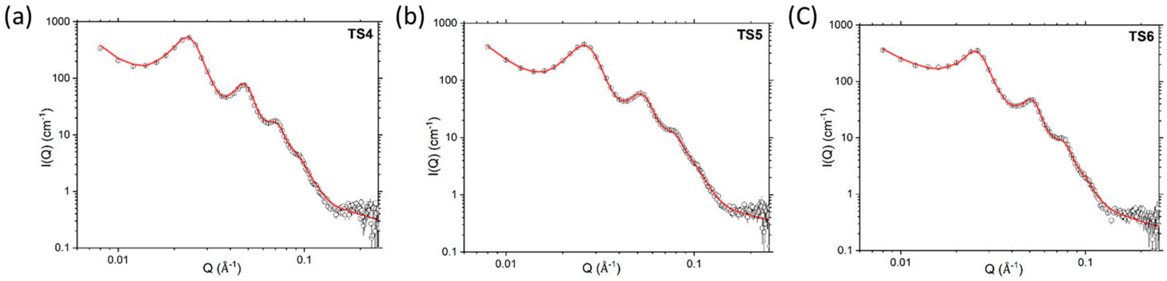

Figure S5. Model-fitted SANS (red line; Model A1) profiles recorded at 318 K for 10% ternary systems prepared with a 1:1 (**a**; C4), 1:0 (b; C5) or 0:1 (c; C6) hexadecanol:octadecanol co-surfactant ratio. Fitted parameters gave rise to a *d*-spacing of 258, 231 and 240 Å and bilayer thicknesses 48 Å, 45 Å and 49 Å for TS4 (a), TS5 (**b**) and TS6 (**c**), respectively. Error bars on the measured data are subsumed within the plotted symbols. Formulation compositions are given in Methods, Table 1.

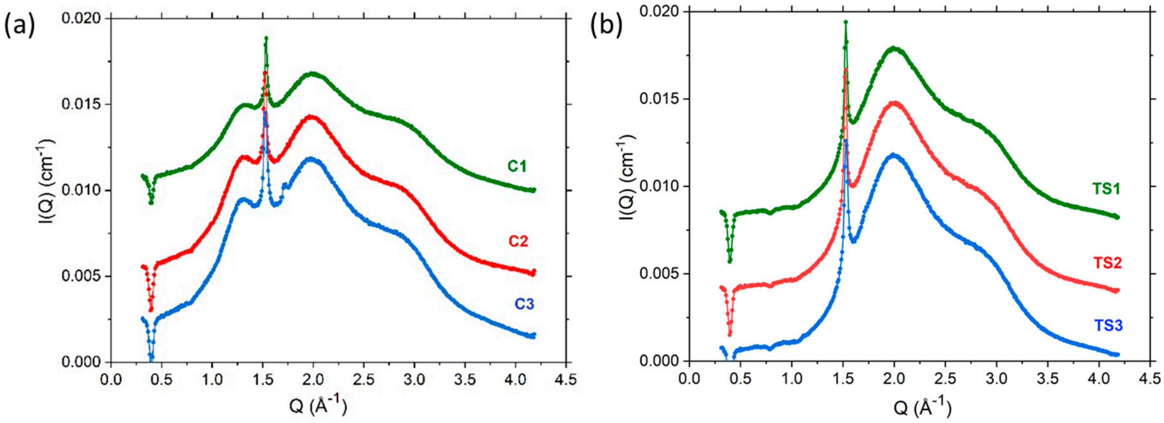

Figure S6. WAXS profiles (offset on the ordinate for clarity) of 4% emulsifier creams (**a**) and ternary systems (**b**) prepared with a 1:1 (green; C1), 1:0 (red; C2) or 0:1 (blue; C3) hexadecanol:octadecanol co-surfactant ratio. Formulation compositions are given in Methods, Table 1.
